# Porcine cysticercosis (*Taenia solium* and *Taenia asiatica*): mapping occurrence and areas potentially at risk in East and Southeast Asia

**DOI:** 10.1186/s13071-018-3203-z

**Published:** 2018-11-29

**Authors:** Uffe Christian Braae, Nguyen Manh Hung, Fadjar Satrija, Virak Khieu, Xiao-Nong Zhou, Arve Lee Willingham

**Affiliations:** 1 One Health Center for Zoonoses and Tropical Veterinary Medicine, Ross University School of Veterinary Medicine, Basseterre, Saint Kitts and Nevis; 2 Department of Parasitology, Institute of Ecology and Biological Resources, Vietnam Academy of Science and Technology, Hanoi, Vietnam; 3 Department of Animal Infectious Diseases, Faculty of Veterinary Medicine, Bogor Agricultural University, Bogor, Indonesia; 4 National Center for Parasitology, Entomology and Malaria Control, Ministry of Health, Phnom Penh, Cambodia; 5 National Institute of Parasitic Diseases, China CDC, Shanghai, China

**Keywords:** *Taenia solium*, *Taenia asiatica*, Mapping, Tapeworm, Neglected Tropical Disease (NTD), Zoonosis, Pigs, Cysticercosis, Taeniosis, East and Southeast Asia

## Abstract

**Background:**

Due to the relative short life span and the limited spatial movement, porcine cysticercosis is an excellent indicator of current local active transmission. The aim of this study was to map at province-level, the occurrence of *T. solium* and *T. asiatica* in pigs and areas at risk of transmission to pigs in East and Southeast Asia, based on the density of extensive pig production systems and confirmed reports of porcine cysticercosis.

**Methods:**

This study covered East and Southeast Asia, which consist of the following countries: Brunei, Cambodia, China, East Timor, Indonesia, Japan, Laos, Malaysia, Mongolia, Myanmar, North Korea, Philippines, Singapore, South Korea, Thailand and Vietnam. Literature searches were carried out to identify current epidemiological data on the occurrence of porcine cysticercosis caused by *T. solium* and *T. asiatica* infections. Modelled densities of pigs in extensive production systems were mapped and compared to available data on porcine cysticercosis.

**Results:**

Porcine cysticercosis was confirmed to be present during the period 2000 to 2018 in eight out of the 16 countries included in this study. *Taenia solium* porcine cysticercosis was confirmed from all eight countries, whereas only one country (Laos) could confirm the presence of *T. asiatica* porcine cysticercosis. Province-level occurrence was identified in five countries (Cambodia, Indonesia, Laos, Myanmar, and Vietnam) across 19 provinces. Smallholder pig keeping is believed to be widely distributed throughout the region, with greater densities predicted to occur in areas of China, Myanmar, Philippines and Vietnam.

**Conclusions:**

The discrepancies between countries reporting taeniosis and the occurrence of porcine cysticercosis, both for *T. solium* and *T. asiatica*, suggests that both parasites are underreported. More epidemiological surveys are needed to determine the societal burden of both parasites. This study highlights a straightforward approach to determine areas at risk of porcine cysticercosis in the absence of prevalence data.

**Electronic supplementary material:**

The online version of this article (10.1186/s13071-018-3203-z) contains supplementary material, which is available to authorized users.

## Background

Pigs can become infected with *Taenia solium* or *Taenia asiatica* if eggs excreted from human tapeworm carriers are ingested. Although, both parasites cause human health concerns, only *T. solium* causes neurocysticercosis, which is a major public health burden globally [1, 2]. In addition, both parasites have an economic impact on livestock sectors. *Taenia solium* and *T. asiatica* share the same transmission pathways from humans to pigs, and back to humans again. Parasite transmission can therefore be addressed with similar intervention approaches.

*Taenia solium* is present throughout the Americas [3], sub-Saharan Africa [4], and believed to be widely endemic in East and Southeast Asia [5]. However, information on the distribution of porcine cysticercosis within East and Southeast Asia, whether due to *T. solium* or *T. asiatica*, is sparse. Due to the relative short life span and the limited spatial movement, porcine cysticercosis infected pigs are excellent indicators of current local active transmission. Keeping pigs in extensive production systems whereby access to human waste is more accessible compared to intensive production systems, is considered the main risk factor for porcine cysticercosis in many regions [6]. The presence of pigs in extensive systems could therefore be used as a first indicator of areas at risk of porcine cysticercosis transmission [3]. The aim of this study was to map, at first-level administrative subdivision level, the occurrence of *T. solium* and *T. asiatica* in pigs and areas at risk of transmission to pigs in East and Southeast Asia, based on the occurrence of smallholder pig production systems and confirmed reports of porcine cysticercosis (*T. solium* and *T. asiatica*).

## Methods

### Study area

This study covered East and Southeast Asia, which consist of the following countries: Brunei (Negara Brunei Darussalam), Cambodia, China, East Timor, Indonesia, Japan, Laos, Malaysia, Mongolia, Myanmar, North Korea, Philippines, Singapore, South Korea, Thailand and Vietnam.

### Data extraction from published and grey literature

Literature searches were carried out to identify epidemiological field surveys or routine slaughter surveys with current data (from year 2000) on the occurrence of porcine cysticercosis caused by *T. solium* and *T. asiatica* infections in East and Southeast Asia. Thus, porcine cysticercosis in this paper only concerns *T. solium* and *T. asiatica* unless otherwise stated. We performed a literature search using PubMed (http://www.ncbi.nlm.nih.gov/pubmed/) with a date restriction from 1st January 2000 to 30th June 2018, and the search term: (“*Taenia solium*” OR “*Taenia asiatica*” OR “swine cysticercosis” OR “porcine cysticercosis” OR “pig cysticercosis”) AND (“Brunei Darussalam” OR “Cambodia” OR “China” OR “East Timor” OR “Hong Kong” OR “Indonesia” OR “Japan” OR “Laos” OR “Macau” OR “Malaysia” OR “Mongolia” OR “Myanmar” OR “Burma” OR “Korea” OR “Philippines” OR “Singapore” OR “Taiwan” OR “Thailand” OR “Vietnam”). The full search output is available in Additional file 1: Table S1. During the initial search phase, titles and abstracts were screened followed by full text review, based on the following exclusion criteria: (i) not dealing with porcine cysticercosis (*T. solium* or *T. asiatica*); (ii) no geographical reference provided; (iii) based on experimental studies or solely on questionnaire surveys; and (iv) data reported were from prior 2000. No language restrictions were imposed. Only the baseline data from studies testing intervention approaches were extracted. Due to the cross-reaction with *Taenia hydatigena* when using serology, data based on serological testing were omitted if data from tongue or carcass inspection from the same country were available. Country reports of porcine cysticercosis (*T. solium*) presence (i.e. “disease present” or “limited distribution”) in the period 2005–2017 were also extracted from the OIE database WAHID Interface [7].

**Fig. 1:**
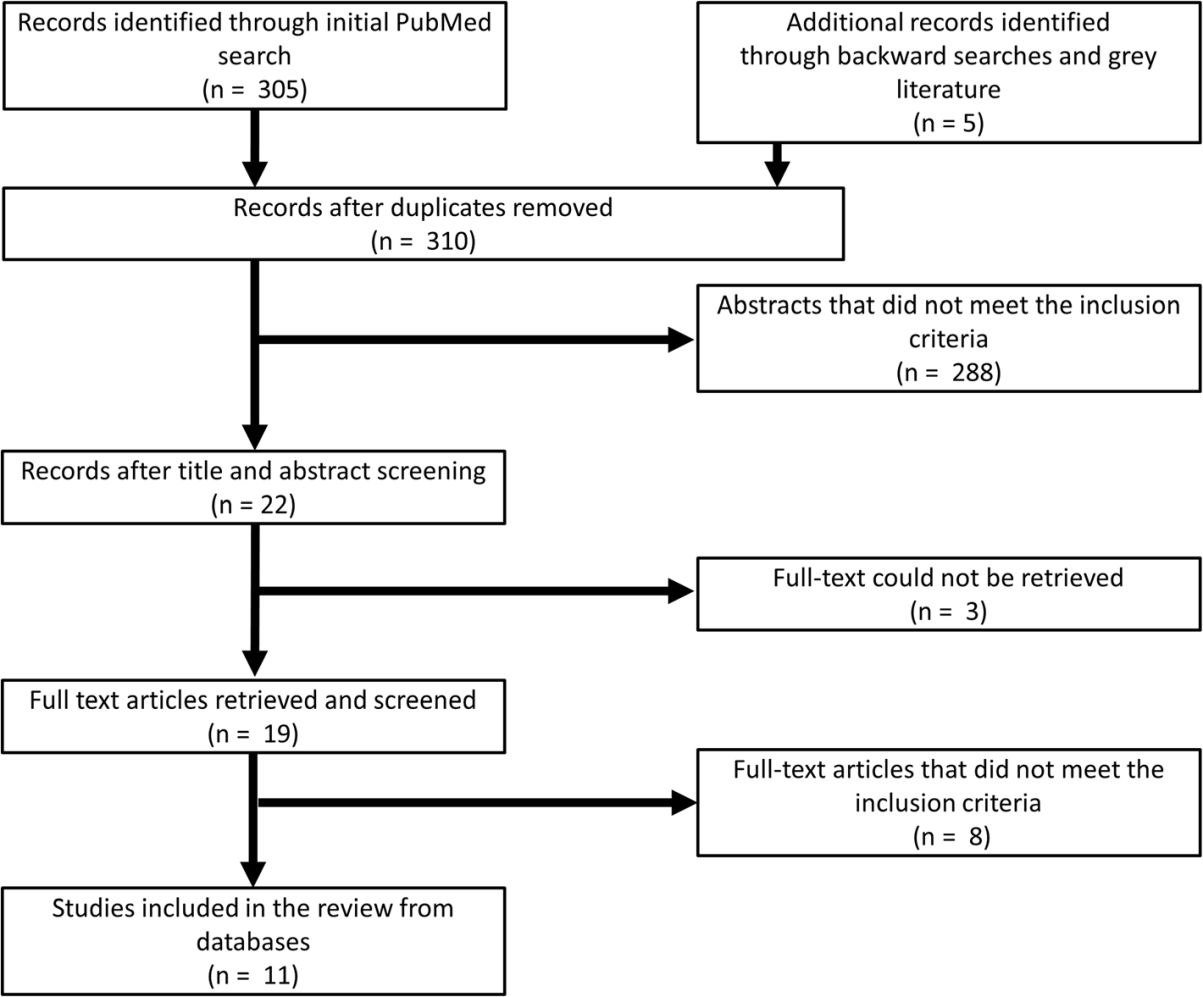
Flow diagram of the search strategy for epidemiological studies on *Taenia solium* or *Taenia asiatica* in pigs

During the second phase, backward reference searches for potential references of epidemiological studies reporting data from 2000–2018 in any of the target countries were conducted on all review articles and eligible articles found during the first search. Collaborators and known researchers from the respective countries were contacted in efforts to obtain grey literature containing epidemiological data from the requested period. Figure 1 shows a flow diagram of the applied search strategies.

In the third phase, if no data on porcine cysticercosis could be obtained from a respective target country, a country-specific PubMed search was carried out. The country-specific search was performed to confirm the presence of the parasites in human hosts within the respective country during the study period by obtaining reports of human cysticercosis, *T. solium* taeniosis, or *T. asiatica* taeniosis, respectively. The country-specific search was performed using the same date restriction as previously and using the term, e.g. (“neurocysticercosis” OR “cysticercosis” OR “taeniosis” OR “taeniasis” OR “*Taenia solium*” OR “*Taenia asiatica*”) AND (“Country name”). Only the newest reports of human cysticercosis, *T. solium* taeniosis and *T. asiatica* taeniosis cases were retained, and the data only used as national level confirmation of presence if the authors provided sufficient evidence to conclude the cases were autochthonous. Furthermore, taeniosis cases were only included if infection was determined to the species level.

### Pigs in extensive production systems

The distribution of pigs raised in extensive production systems (smallholder pig keeping) was mapped using geographical information systems based on modelled data available through http://livestock.geo-wiki.org [8, 9].

## Results

**Fig. 2:**
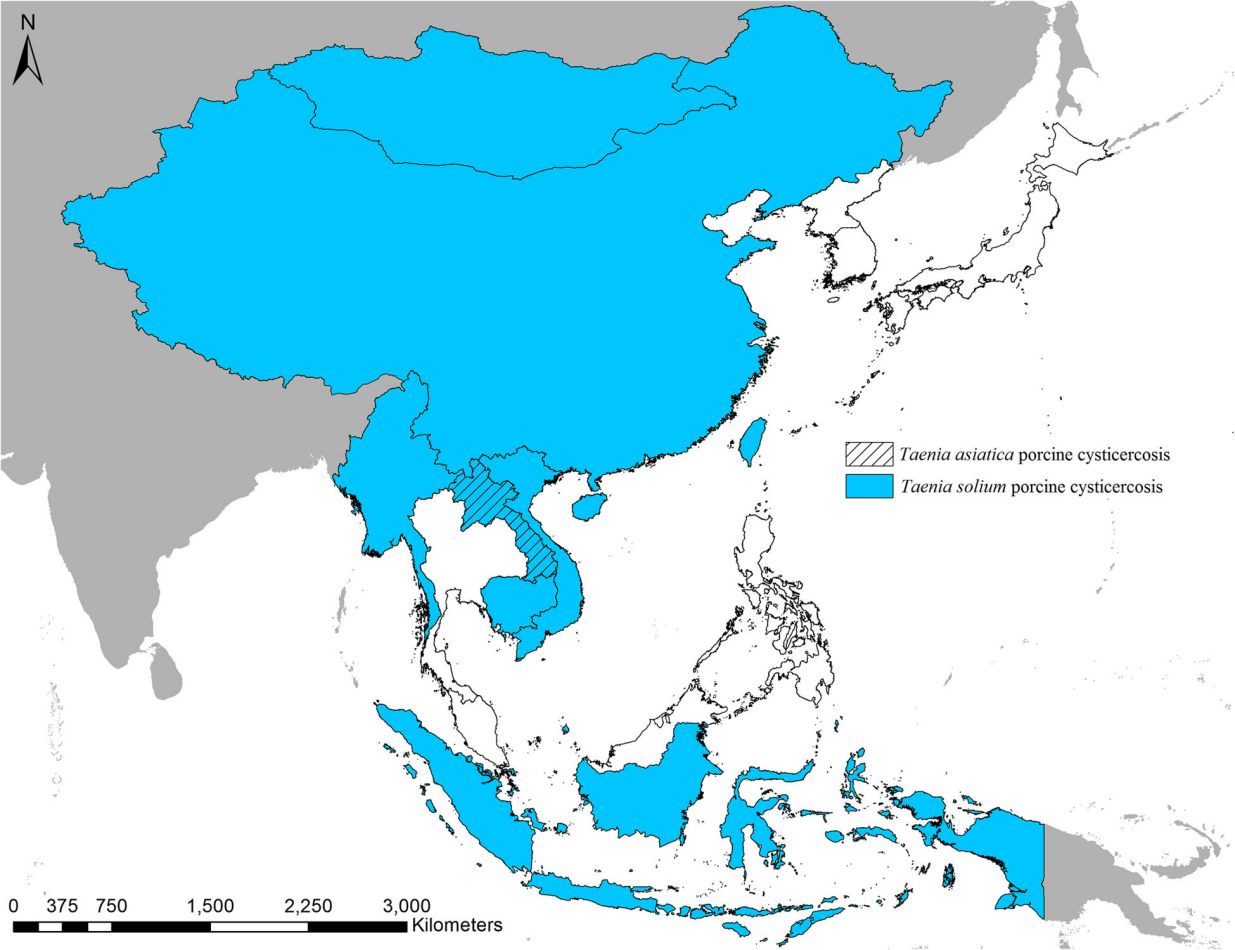
Countries in East and Southeast Asia with confirmed reports of porcine cysticercosis (*Taenia solium*/*Taenia asiatica*) based on *post-mortem* data from the period January 1st 2000 to June 30th 2018

The searchers revealed 310 reports, of which only 11 reports contained sufficient epidemiological data to be considered in the study. Porcine cysticercosis was confirmed to be present in eight out of the 16 countries included in this study during the period 2000 to 2018 (Table 1, Fig. 2). *Taenia solium* porcine cysticercosis was confirmed from all eight countries, whereas only one country (Laos) could be confirmed for the presence of *T. asiatica* porcine cysticercosis. In addition, of the eight countries with porcine cysticercosis, five were confirmed to have occurrence of *T. solium* porcine cysticercosis *via* the OIE database [7], and for two of those five countries (China and East Timor), OIE was the only data source confirming the occurrence of *T. solium* porcine cysticercosis that could be obtained (Table 1). No national level occurrence of porcine cysticercosis were confirmed solely based on serology. Eleven studies were identified to provide epidemiological data on porcine cysticercosis, and eight of them had sufficient data to provide information on province level distribution (Additional file 1: Table S2).

**Table 1:** List of reports with confirmed cases of *Taenia solium* or *Taenia asiatica* in pigs and humans in the period January 1st 2000 to June 30th 2018. Prevalence has not been included as samples are not representative, but the parasite noted as being present within a specified area based on sufficient evidence. Taeniosis and human cysticercosis data have only been included if no reports of positive pigs could be found to illustrate potential presence of the parasite within the area and only the most recent data have been included

Country	*T. solium*			*T. asiatica*	
	Porcine cysticercosis	Taeniosis	Human cysticercosis	Porcine cysticercosis	Taeniosis
Brunei	–	–	–	–	–
Cambodia	Meat inspection of 432 pigs (29 infected) [35]	na	na	–	–
China	OIE reported cases occurring during 2005–2017	na	na	–	+ [18]
East Timor	OIE reported cases occurring during 2005–2017	na	na	–	–
Indonesia	Meat inspection of 35 pigs (27 infected) [36]. *Post-mortem* examination of an infected pig [37]. *Post-mortem* examination of 3 pigs (1 infected) [38]. Meat inspection of 6 seropositive pigs (6 infected) [39]	na	na	–	+ [21]
Japan	–	–	–	–	+ [22]
Laos	Meat inspection of 590 pigs (5 infected) [10]. OIE reported cases occurring during 2005–2017	na	na	Meat inspection of 590 pigs (1 infected) [10]	na
Malaysia	–	–	–	–	–
Mongolia	OIE reported cases occurring during 2005–2017	na	na	–	–
Myanmar	Meat inspection of 300 pigs (71 infected) [40]	na	na	–	–
North Korea	–	–	+ [27]	–	–
Philippines	–	–	+ [28]	–	–
Singapore	–	–	–	–	–
South Korea	–	–	–	–	+ [32]
Thailand	–	+ [33]	+ [33]	–	+ [33]
Vietnam	Tongue examination of 172,087 pigs (109 infected) [41]. OIE reported cases occurring during 2005–2017	na	na	–	+ [12]

*Abbreviation*: *na* not available

**Fig. 3:**
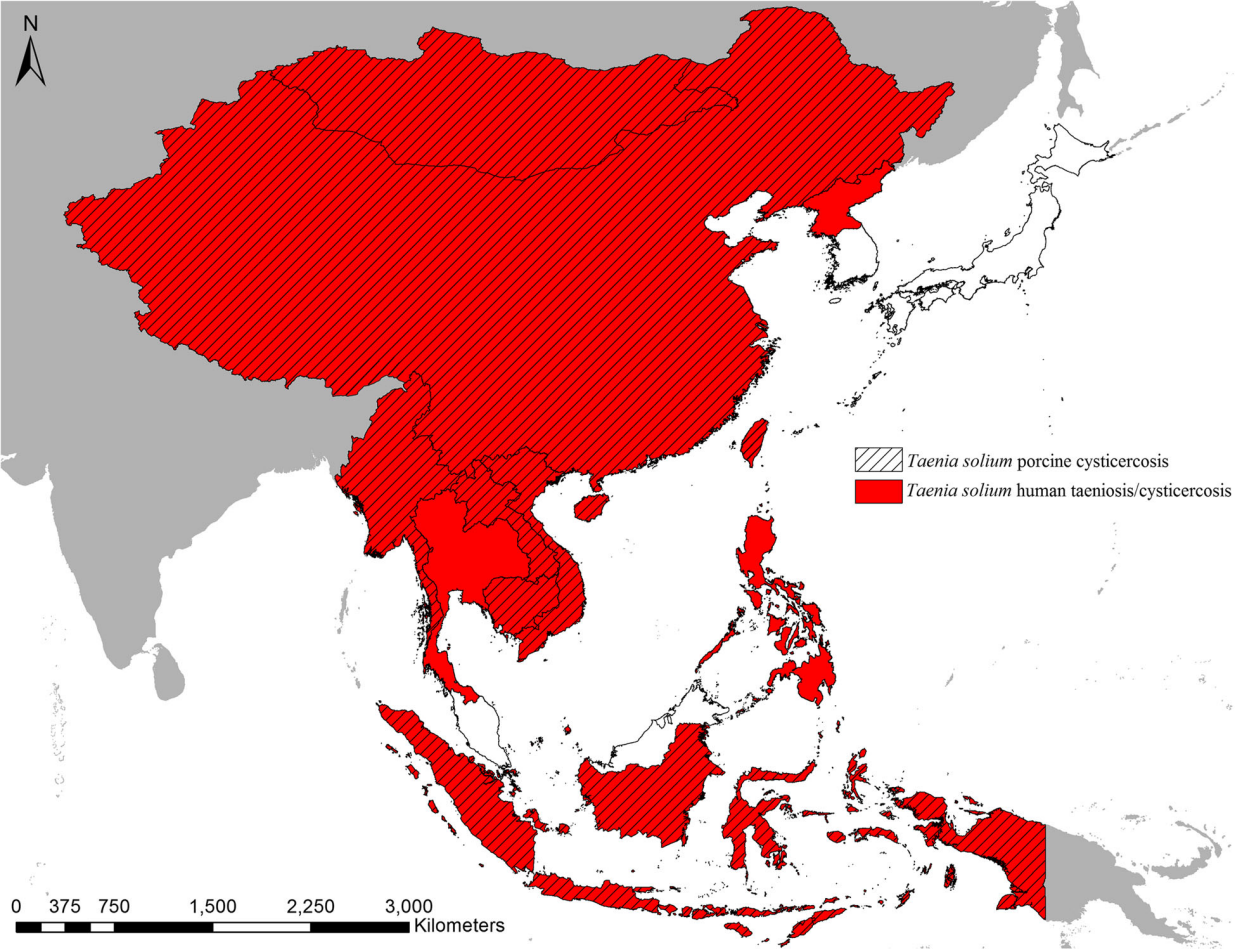
Countries in East and Southeast Asia with confirmed reports of *Taenia solium* in humans and specifically reports of *T. solium* porcine cysticercosis in the period January 1st 2000 to June 30th 2018

**Fig. 4:**
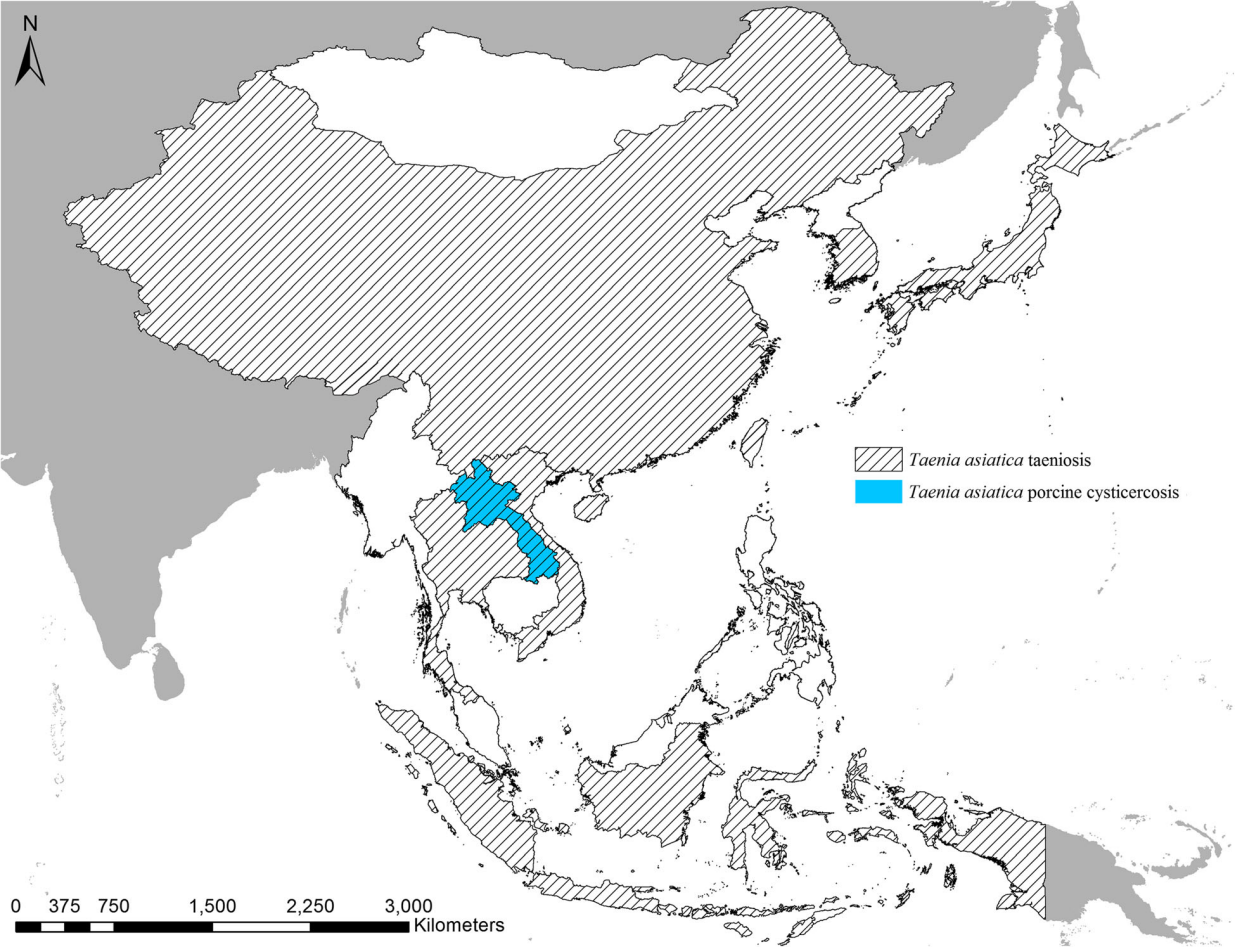
Countries in East and Southeast Asia with confirmed reports of *Taenia asiatica* taeniosis and *T. asiatica* porcine cysticercosis in the period January 1st 2000 to June 30th 2018

Both *T. solium* taeniosis and *T. solium* cysticercosis in humans could be confirmed in Thailand, whereas only human cysticercosis occurrence was confirmed in North Korea and the Philippines. In total, *T. solium* was confirmed in 11 out of the 16 countries included in this study (Fig. 3). In addition to Laos where porcine cysticercosis was confirmed, *T. asiatica* was also confirmed in six additional countries (China, Indonesia, Japan, South Korea, Thailand and Vietnam) (Fig. 4).

**Fig. 5:**
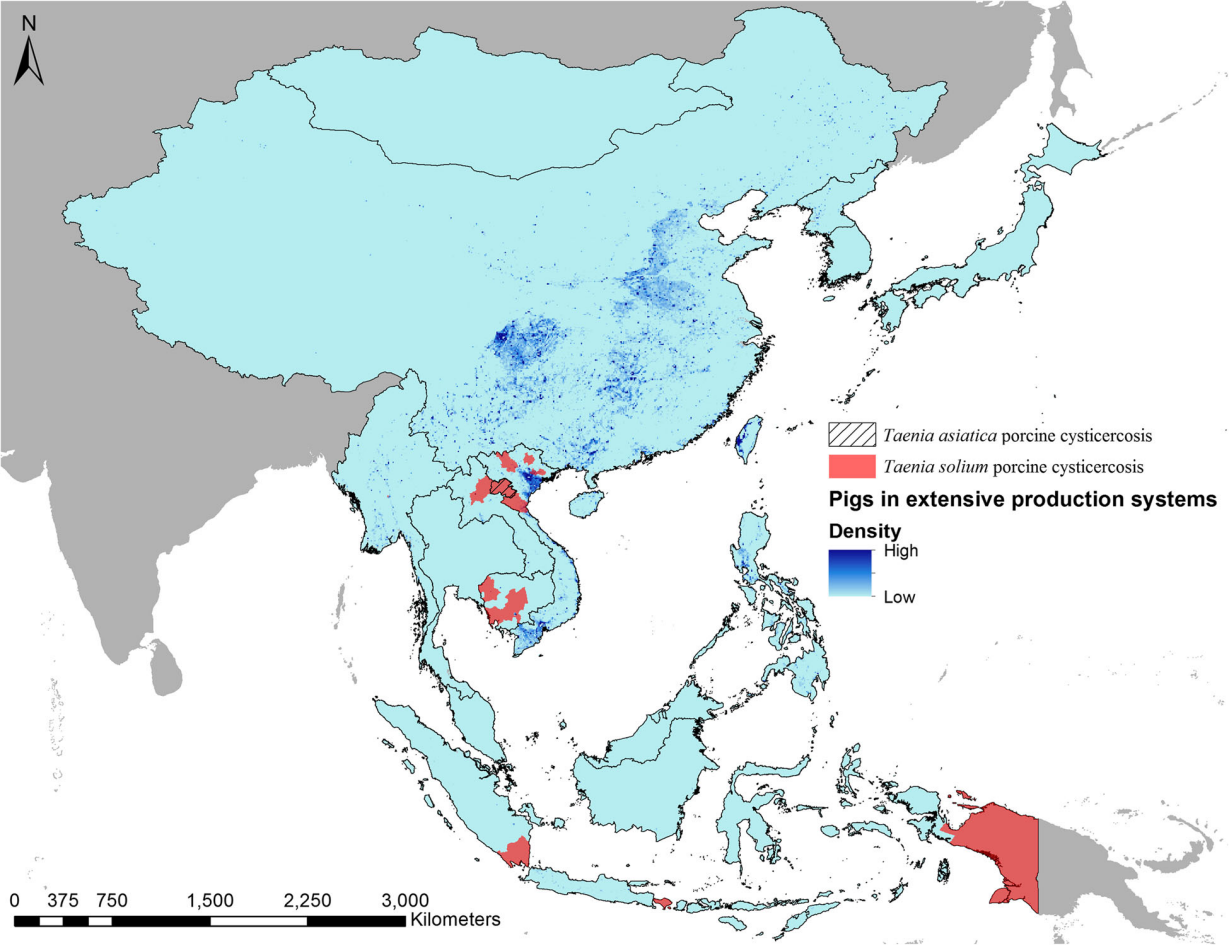
Provinces (first-level administrative subdivision) in East and Southeast Asia with porcine cysticercosis in the period January 1st 2000 to June 30th 2018. Density data for pigs kept in extensive productions systems were extracted from [8, 9]

We identified eight studies where porcine cysticercosis occurrence could be confirmed on a province level (Table 2). One of these studies found both *T. solium* and *T. asiatica* in pigs from Huaphan Province, Laos [10]. Province level occurrence was identified in five countries (Cambodia, Indonesia, Laos, Myanmar and Vietnam) across 19 provinces. Smallholder pig keeping is believed to be widely practised throughout the region, with greater densities predicted to occur in areas of China, Myanmar, Philippines and Vietnam. Figure 5 shows the provinces found to harbour porcine cysticercosis infected pigs, overlaid with the previously modelled density of pigs kept in extensive production systems throughout the region [8, 9].

**Table 2:** The first-level administrative subdivision (province) occurrence of *Taenia solium* porcine cysticercosis in East and Southeast Asia from January 1st 2000 to June 30th 2018 based on *post-mortem* examinations

Country	Reference	First-level administrative subdivision (Province)
Cambodia	[35]	Banteay Mean Chey, Battambang, Kampong Cham, Kampong Chhnang, Kampong Speu, Kampong Thom, Kandal, Koh Kong
Indonesia	[36–39]	Papua, Bali, Lampung
Laos	[10]	Luangprabang, Huaphan^a^
Myanmar	[40]	Naypyidaw
Vietnam	[41]	Yen Bai, Lao Cai, Nghe An, Bac Kan, Bac Giang

^a^*Taenia asiatica* also confirmed

## Discussion

On a national level, of the 16 countries covering East and Southeast Asia, eight were found to have confirmed reports of *T. solium* porcine cysticercosis and only Laos had confirmed reports of pigs being infected with *T. asiatica* during the study period. Discrepancy in reports of *T. solium* porcine cysticercosis and reports of infections in humans occurred as only human infections were observed for three countries (North Korea, Philippines and Thailand). This is most likely a consequence of epidemiological surveys in pigs not having been performed. The results of this study also underlined the discrepancy between the known distribution of pigs infected with *T. asiatica*, which were only reported from one province in Laos, and human *T. asiatica* taeniosis cases, which were reported from an additional six countries. This indicates that *T. asiatica* porcine cysticercosis is vastly underreported, and that more epidemiological survey data from across the region are required in order to determine the burden of this parasite within East and Southeast Asia.

Although porcine cysticercosis and taeniosis were reported throughout the region, little data were available to confirm the distribution of porcine cysticercosis on a provincial level (first-administration subdivision level). Provinces with infected pigs were identified in northern Laos and Vietnam, central Myanmar, southern Laos and Sumatra, Bali and Papua. Using modelled distribution data of the density of pigs in extensive production systems can provide a cost-effective approach to map out areas potentially at risk of porcine cysticercosis. The majority of pigs kept in extensive systems were predicted to be found in China. According to the global burden of disease study in 2013, China has the second most reported cases of human cysticercosis globally [11], but epidemiological studies or data on porcine cysticercosis originating from China are unavailable to the international research community. Higher densities of pigs in extensive systems were also predicted in Myanmar, southern and northern Vietnam, the Philippines and China. Modelling the distribution of pigs is limited by the quality of the underlying data. Studies are still missing to determine to which degree risk of porcine cysticercosis can be correlated with the density of pigs in extensive production systems. However, the modelled distribution provides an initial approach to identify areas where epidemiological data will most likely be required to accurately model the regional distribution of porcine cysticercosis.

With increased international travel and movement of people across borders, case reports of taeniosis are not necessarily reliable to confirm active transmission of the parasite from a specific location, which requires more costly epidemiological surveys. However, a newly developed assay based on multiplex qPCR on stool samples appears to be able to distinguish between the three species of *Taenia* tapeworms (*T. asiatica*, *T. saginata* and *T. solium*) causing taeniosis in humans [12]. Available data on taeniosis where no species identification has been made is not useful in the determination of burden of the various parasites. Relying on porcine cysticercosis surveillance to establish parasite presence is justifiable and an excellent indicator of active transmission due to pigs’ relative short lifespan and limited distance in geographical movement. However, surveillance of porcine cysticercosis also has diagnostic complications. No national level occurrences of porcine cysticercosis were confirmed solely based on serology in this study. Serological confirmation of porcine cysticercosis, if there is a presence of *T. hydatigena*, becomes highly problematic due to cross-reactions in the assays. The reported prevalence of *T. hydatigena* in Asia has been relatively high [13] compared to reports from Africa [14], which underlines the importance of confirming porcine cysticercosis based on lingual or* post-mortem* examinations of pigs.

### Brunei Darussalam

We were not able to identify any data to suggest that porcine cysticercosis is a problem in Brunei Darussalam. Nor was any other literature found that could indicate the presence of either *T. solium* or *T. asiatica* taeniosis in the human population. The population in Brunei is predominantly Muslim and therefore do not consume pork, potentially the primary cause of the pathogens absence. The need for further investigations in Brunei currently seems unwarranted.

### Cambodia

As illustrated in Fig. 5, porcine cysticercosis due to *T. solium* is widely endemic in Cambodia. However, the data available were based on limited sample sizes from the affected provinces and more epidemiological surveys are therefore necessary to establish the degree of endemicity with the country. Cambodian schoolchildren have been found infected with *Taenia* spp. [15], but reports of human taeniosis due to *T. solium* or *T. asiatica* are not well documented from Cambodia. It is not clear to what degree neurocysticercosis is a problem within Cambodia as no information is available, but in a recent study, people were found seropositive for cysticercosis [16]. More detailed mapping of both *Taenia* species is recommended in Cambodia in order to estimate burden and implement control initiatives accordingly.

### China

There are older reports of porcine cysticercosis being a problem throughout China [17], but no current reports of porcine cysticercosis are readily available. Even though no reports of porcine cysticercosis due to *T. asiatica* could be found, there have been recent reports of *T. asiatica* taeniosis in China, suggesting that this parasite might be common in certain areas [18]. More epidemiological data are required to map to extent of the problem in China. A recent epidemiological study of human cysticercosis in the central part of China indicates that *T. solium* is still a major problem within China [19], warranting further surveys to determine the distribution and burden of *T. solium* as well. Chinese Taiwan has previously been endemic for both *T. solium* and *T. asiatica*, but no current information is readily available to describe the status of these two parasites. Epidemiological surveys are highly warranted to estimate the distribution and prevalence on Chinese Taiwan, due to the history of occurrence [5].

### East Timor

OIE reported that *T. solium* porcine cysticercosis occurs in the country, but we were unable to find other reports. No reports of *T. asiatica* in either pigs or humans could be found. The OIE reports need to be followed-up by epidemiological investigations to determine the distribution of *T. solium* porcine cysticercosis in East Timor.

### Indonesia

The reports of porcine cysticercosis from Indonesia are few, but it is also likely that transmission of *T. solium* to pigs is highly clustered and restricted to areas where Islam is not practiced predominantly. This study provides a rough overview of areas that should be considered for epidemiological surveys to establish the extent of *T. solium* distribution throughout Indonesia. *Taenia asiatica* has not been reported in pigs from any recent studies, but there have been reports of *T. asiatica* found in the livers of pigs in the past [20]. More recent reports of *T. asiatica* taeniosis exist, but not directly published yet [21].

### Japan

There are no recent reports of porcine cysticercosis in Japan, and the country is not considered to have active transmission of porcine cysticercosis. However, recently human *T. asiatica* taeniosis cases have been confirmed within Japan [22]. In addition, imported cases of taeniosis can still occur and cause sporadic outbreaks of cysticercosis. Recently, an imported case of *T. asiatica* taeniosis believed to have originated in the Philippines was reported in Japan [23].

### Laos

Recently all three species, *T. solium*, *T. saginata* and *T. asiatica*, were found in people living in Laos [24]. The study also reported a number of people being seropositive for cysticercosis. Together, these are clear indications that active transmission of both *T. solium* and *T. asiatica* presently occurs in Laos. However, more epidemiological data are needed to establish in which parts of the country transmission of porcine cysticercosis occurs frequently.

### Malaysia

There are no current reports of porcine cysticercosis in Malaysia where the predominant religion is Islam. Older studies have indicated that rural areas populated with communities not belonging to Islam, have been exposed to *T. solium*. In 1996, 135 blood samples collected were analysed using an Ab-ELISA and a prevalence of 2.2% was found [25].

### Mongolia

OIE reported cases of *T. solium* porcine cysticercosis occurring within Mongolia. However, no reports of *T. solium* infections in humans were found nor were any reports of *T. asiatica*. Epidemiological surveys should be performed in the areas reported to the OIE to estimate the extent of the *T. solium* porcine cysticercosis distribution in Mongolia.

### Myanmar

*Taenia solium* porcine cysticercosis was confirmed from Myanmar and the presences of *T. asiatica* have been suggested to occur, but so far, there are no published reports of infected pigs based on *post-mortem* examination from Myanmar. Recently both people and pigs have been found seropositive for cysticercosis as well as people positive for taeniosis [26].

### North Korea

Very limited information about the presence of disease exists from North Korea, but one study based on serology in humans suggest that *T. solium* is actively transmitted in North Korea [27]. There are no reports of *T. asiatica* from North Korea. Whether porcine cysticercosis is endemic in North Korea remains unknown, and epidemiological surveys do not seem to be implementable in the near future.

### Philippines

Although there were no recent reports of porcine cysticercosis from the Philippines, a recent community survey found a seroprevalence among humans of almost 25% based on antibodies [28], which is a strong indicator of *T. solium* occurrence. A presumed imported case of *T. asiatica* taeniosis in Japan, based on the patient’s travel history, was believed to have originated in the Philippines [23]. This could indicate that *T. asiatica* is still endemic in certain parts of the Philippines, but no current information is available.

### Singapore

Singapore is not considered endemic for porcine cysticercosis, but does receive reports occasionally of human cysticercosis, presumably due to the influx of foreign travellers and workers [29]. However, a substantial amount of pork is consumed annually in Singapore, but pork production is minimal. Singapore imports frozen or processed pork from numerous countries, but until recently the only source of fresh pork was from Riau Islands Province, Indonesia. Import of live pigs from Malaysia has recently commenced after being banned in 1999 due to a Nipah Virus outbreak [30]. This could increase the risk of importing cases of porcine cysticercosis into the country, but the risk of active transmission in Singapore is minimal.

### South Korea

Cysticercosis is not considered endemic in South Korea [31]. However, a recent case report of *T. asiatica* taeniosis showed that sporadic transmission is still suspected within South Korea [32]. Epidemiological surveys of pigs in rural areas are needed to confirm or refute the presence of porcine cysticercosis in South Korea.

### Thailand

Even though no data exist for pigs, a community-based survey conducted in two villages in central Thailand during 2007 and 2008, reported both the presence of *T. solium* (taeniosis and cysticercosis) and *T. asiatica* in the human population [33]. Endemic foci may exist throughout the country, and the study by Anantaphruti et al. [33] warrants further investigations in the porcine populations.

### Vietnam

OIE and epidemiological surveys confirm the presence of *T. solium* in Vietnam. Data describing the distribution are still very limited. Although no recent reports of *T. asiatica* porcine cysticercosis could be found from Vietnam, there have been several reports of taeniosis cases, recently reviewed by Ng-Nguyen et al. [34]. Studies are needed to map the distribution and determine the burden of *T. solium* and *T. asiatica* within Vietnam.

## Conclusions

A common feature for all countries is that the detailed national distributions of porcine cysticercosis are missing. The discrepancies between countries reporting taeniosis and the occurrence of porcine cysticercosis agents, both *T. solium* and *T. asiatica*, suggest that both parasites are underreported. More should be done to uncover the distribution of porcine cysticercosis caused by *T. solium* and *T. asiatica* in East and Southeast Asia. This will provide researchers and stakeholders with the opportunity to estimate the burden imposed by these two parasites on societies within the region. We have here attempted to provide a rough estimate of the areas at risk of porcine cysticercosis based on the density of pigs kept by smallholder farmers. Future epidemiological surveys will be able to answer to what degree this approach can be used to determine which areas to include in control initiatives against not only *T. solium*, but also against *T. asiatica*. Due to the paucity of epidemiological data, the approach taken here to risk mapping is an initial start.

## Additional file

Additional file 1: **Table S1.** Full search output from the initial literature search. **Table S2.** List of studies that were identified to provide epidemiological data on porcine cysticercosis. (XLSX 45 kb)
